# The efficacy of thoracolumbar interfascial plane block for lumbar spinal surgeries: a systematic review and meta-analysis

**DOI:** 10.1186/s13018-023-03798-2

**Published:** 2023-04-25

**Authors:** Guanghua Long, Chong Liu, Tuo Liang, Xinli Zhan

**Affiliations:** Spine and Osteopathy Ward, The First Affiliated Hospital of Guangxi Medical University, 6 Shuangyong Road, Nanning, Guangxi China

**Keywords:** Regional anesthesia, Analgesia, Spinal surgery, Pain

## Abstract

**Background:**

The intent of this meta-analysis was to examine the efficacy of thoracolumbar interfascial plane block (TLIP) for pain control after lumbar spinal surgery.

**Methods:**

Randomized controlled trials (RCTs) published on PubMed, CENTRAL, Scopus, Embase, and Web of Science databases up to February 10, 2023, comparing TLIP with no or sham block or wound infiltration for lumbar spinal surgeries were included. Pain scores, total analgesic consumption, and postoperative nausea and vomiting (PONV) were analyzed.

**Results:**

Seventeen RCTs were eligible. Comparing TLIP with no block or sham block, the meta-analysis showed a significant decrease of pain scores at rest and movement at 2 h, 8 h, 12 h, and 24 h. Pooled analysis of four studies showed a significant difference in pain scores at rest between TLIP and wound infiltration group at 8 h but not at 2 h, 12 h, and 24 h. Total analgesic consumption was significantly reduced with TLIP block as compared to no block/sham block and wound infiltration. TLIP block also significantly reduced PONV. GRADE assessment of the evidence was moderate.

**Conclusion:**

Moderate quality evidence indicates that TLIP blocks are effective in pain control after lumbar spinal surgeries. TLIP reduces pain scores at rest and movement for up to 24 h, reduces total analgesic consumption, and the incidence of PONV. However, evidence of its efficacy as compared to wound infiltration of local anesthetics is scarce. Results should be interpreted with caution owing low to moderate quality of the primary studies and marked heterogeneity.

**Supplementary Information:**

The online version contains supplementary material available at 10.1186/s13018-023-03798-2.

## Introduction

Recent advances in minimally invasive procedures and bone healing strategies have improved outcomes of patients undergoing lumbar spinal surgeries [1]. However, considering the complex anatomy and invasive nature of the surgery, pain control is of utmost importance. Spinal surgeries are often accompanied by excessive pain due to extensive dissection and muscle retraction during the procedure. Inadequate pain control not only delays patient recovery and rehabilitation but is also an important source of patient dissatisfaction [2].

Non-steroidal anti-inflammatory drugs and more commonly opioids are central to pain management post-lumbar spinal interventions. But opioids are not only related with inherent adverse-effects like postoperative nausea and vomiting (PONV), delirium, sedation, constipation, tolerance, respiratory depression, etc., [3] but also have been recently correlated with increased risk of reoperations post-lumbar surgeries [4]. Studies have noted high dependence of opioid drugs among patients undergoing spinal procedures [5]. Therefore, these has been an concerned effort to reduce dependence on opioids and increase multimodal analgesic modalities after spinal surgeries. Among these, the enhanced recovery pathways have been successfully tried for lumbar spinal procedures with an aim to reduce length of hospital stay and reduce opioid requirements [6]. Since one of the components of enhanced recovery pathway is use of regional anesthesia, it is imperative that new methods of regional nerve blocks are developed to improve the postoperative course with minimal use of opioids.

In 2015, the thoracolumbar interfascial plane block (TLIP) was first proposed by Hand et al. to provide midline anesthesia for spinal surgeries [7]. Since then, the TLIP has been studies by a number of studies and even reviewed by two recent meta-analyses [8, 9]. However, these reviews could include only nine studies each with further reduced quantity of studies in the meta-analysis. Furthermore, in view of a retracted trial [10] and publication of further studies [11–13] in the recent past there is a need for a comprehensive updated meta-analysis which provides accurate and reliable evidence on the efficacy of TLIP for postoperative analgesia after lumbar spinal surgeries. Hence, the current study was undertaken to compare the analgesic efficacy of TLIP vs control or wound infiltration in lumbar surgery patients.

## Material and methods

### Search

The protocol with all study objectives were registered on PROSPERO (CRD42023396349) before beginning the search. An elaborate search of PubMed, CENTRAL, Scopus, Embase, and Web of Science was undertaken. Gray literature was additionally searched using Google Scholar and Open Gray (http://www.opengrey.eu). www.clinicaltrials.gov was searched for any ongoing trials with published results. The search date was concluded on February 10, 2023. Search terms were: “spine”, “spinal”, “lumbar”, “lumbar surgery”, “thoracolumbar interfascial plane block”, “TLIP”, “analgesia”, and “randomised controlled trial”. The common search plan is shown in Additional file 1: Table S1. The search results were examined by two reviewers separately. Duplicates were excluded and articles were reviewed by titles/abstracts. Relevant studies underwent full-text analysis before inclusion. Disagreements were resolved by discussion. The search was supplemented in the end by examining the reference list of included studies.

### Eligibility

PICOS inclusion criteria were:

Population: Patients undergoing any lumbar spinal surgical procedure.

Intervention: TLIP.

Comparison: No block, sham block, or wound infiltration.

Outcomes: Pain scores, total analgesic consumption post-surgery, and PONV.

Study type: RCTs.

We excluded non-RCTs, trials with overlapping data, review articles, and editorials. There was no language restriction for inclusion in the review. References of previous meta-analyses on the topic were also searched for inclusion of any missing trials.

### Data extraction

Last author, year of publication, study location, type of surgical procedure, the anesthetic agent used, the protocol of the control group, type of postoperative patient-controlled analgesia (PCA), sample size, age of participants, gender details, and outcome data were extracted using a data spreadsheet. In case of incomplete data, corresponding authors were contacted once by email. The review outcomes were pain measured on a 10-point scale in the first 24 h at 2, 8, 12, and 24 h. If trials reported pain scores at 1 h and 6 h, they were included in the analysis of 2 h and 8 h. Total analgesic consumption via PCA in the postoperative period was the second outcome of interest. Lastly, we pooled data on the incidence of PONV.

The risk of bias was judged using the Cochrane Collaboration risk of bias-2 tool [14]. Trials were marked as low or high risk, or some concerns for every domain in the assessment tool. The different domains of the tool included: the randomization process, deviation from intended intervention, missing outcome data, measurement of outcomes, selection of reported results, and overall risk of bias. Grading of Recommendations Assessment, Development, and Evaluation (GRADE) tool based on the GRADEpro GDT software was used to judge the certainty of the evidence.

### Statistical analysis

Pain and analgesic consumption data were extracted as mean and standard deviations (SD). In the case of studies that reported data in the form of median and range or interquartile values, it was transformed by methods described by Wan et al. [15]. In case data were available only in figure format, Engauge Digitizer version 12.1 was used. Pain data being measured on the same scale were combined as mean difference (MD) with 95% confidence intervals (CI). As total analgesic consumption was measured using different opioid drugs or a combination of opioids and other analgesics, data were combined as standardized mean difference (SMD). A separate analysis was conducted for studies using local anesthetic infiltration in the control group. Pain scores at rest and movement were also pooled separately. All analyses were done in a random-effects model.

A sensitivity analysis was done to check the stability of the results. This was carried out by removing one study at a time from the software. The *I*^2^ statistic was the tool to check between-study heterogeneity. Funnel plots were used to check for publication bias. The software used was “Review Manager” (RevMan, version 5.3; Nordic Cochrane Centre [Cochrane Collaboration], Copenhagen, Denmark; 2014). PRISMA reporting guidelines were abided for the review [16].

## Results

### Search and baseline details

**Fig. 1:**
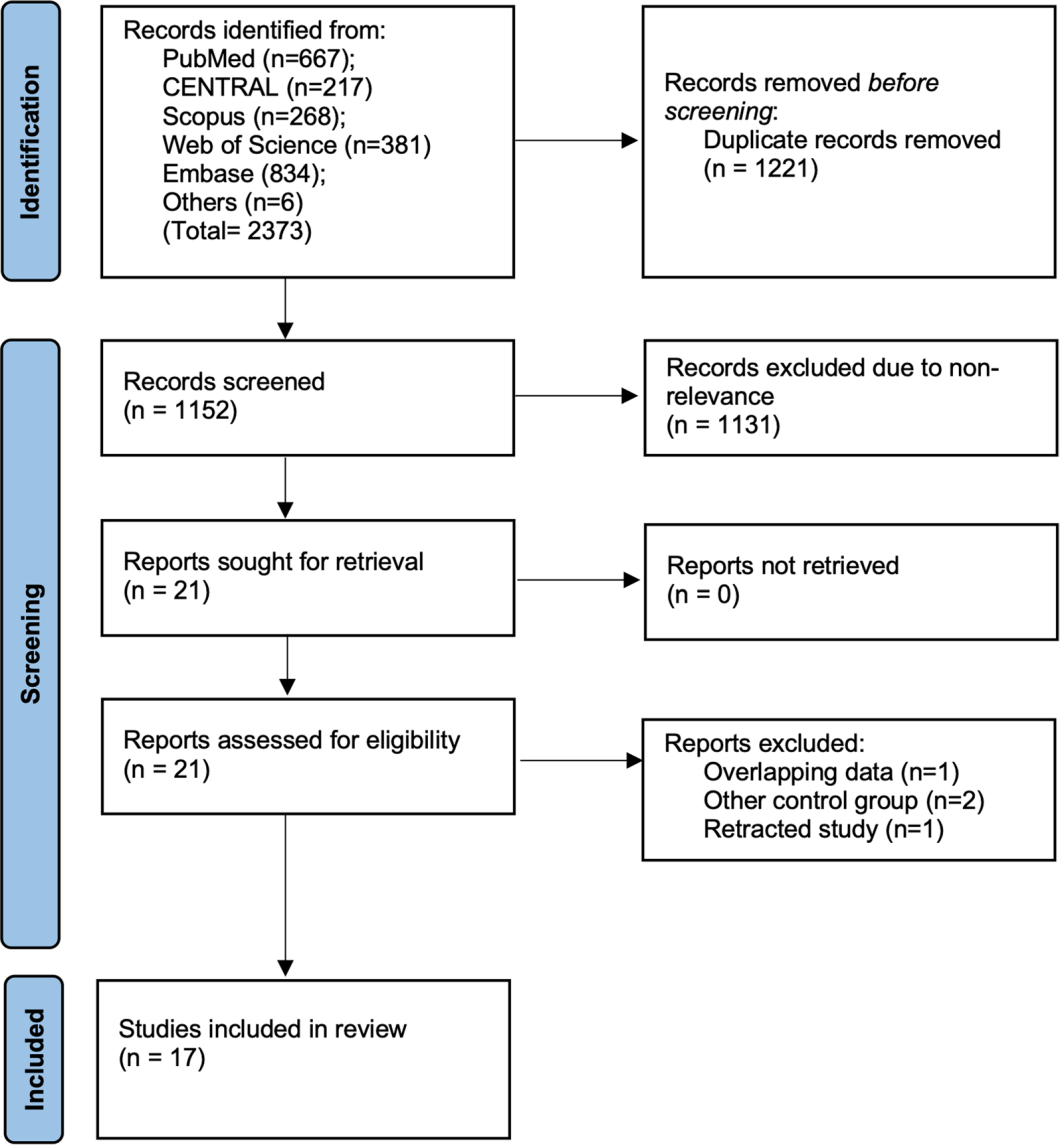
Study flowchart

Two thousand three hundred and seventy three articles were found following the literature search. On deduplication, 1152 of these were unique. On further initial title/abstract screening, 21 were picked for full-text analysis (Fig. 1).

The study details extracted are produced in Table 1. All trials were published recently between 2018 and 2023 and were from Turkey, China, Egypt, or India. There was a predominance of studies from the first two countries. Surgical procedures were done under general anesthesia in all studies and involved lumbar fusion, disc surgeries, and internal fixation. All TLIP blocks were carried out using ultrasonography using either bupivacaine or ropivacaine. In four studies, local anesthetic infiltration of the surgical site was done. One study used a combination of Sufentanil and flurbiprofen for PCA, while most of the others used only sufentanil or fentanyl. The total sample size included in the 17 trials was 1205 with 615 in the study group and remaining in the control group.

**Table 1:** Details of included studies

Study	Location	Surgical procedure	Anesthetic agent for TLIP	Control group	PCA	Groups	Sample size	Mean age	Male gender (%)
Ahiskalioglu [17]	Turkey	Posterior lumbar instrumentation surgery	20 mL 0.25% bupivacaine	Sham block	Fentanyl	Study Control	20 20	NR	NR
Ammar [18]	Egypt	Herniated lumbar disc surgery	20 ml mixture of 0.25% bupivacaine and 1% lidocaine	No block	Morphine	Study Control	35 35	42 43.5	60 65.7
Guo [19]	China	Posterior lumbar spine fusion	20 mL 0.5% ropivacaine	No block	Sufentanil	Study Control	20 20	58 58	30 35
Chen [20]	China	Lumbar spine fusion surgery	20 mL 0.25% bupivacaine	Sham block	Sufentanil	Study Control	30 30	58.6 53.9	26.7 25
Cheng [21]	China	Internal fixation	20 ml of 0.375% ropivacaine	No block	Sufentanil	Study Control	24 24	56.2 56	50 50
Ince [22]	Turkey	Single-level lumbar discectomy	20 mL 0.25% bupivacaine	Wound infiltration	Fentanyl	Study Control	20 20	48.5 44.8	55 50
Li [23]	China	Posterior lumbar fusion and internal fixation	20 ml of 0.375% ropivacaine	No block	Sufentanil	Study Control	25 25	49.4 49.5	48 40
Ozmen [24]	Turkey	Single-level lumbar discectomy	20 mL 0.25% bupivacaine	Sham block	Fentanyl	Study Control	40 40	48.9 44.6	52.5 55
Shi [25]	China	Lumbar spine surgery	20 mL 0.25% ropivacaine	No block	Sufentanil	Study Control	37 37	44 43.1	59.5 62.1
Yu [26]	China	Lumbar spine fusion surgery	20 mL 0.5% ropivacaine	No block	Sufentanil	Study Control	49 24	58 58	42.8 41.7
Ekinci [27]	Turkey	Single-level lumbar discectomy	20 mL 0.25% bupivacaine	Wound infiltration	Fentanyl	Study Control	30 30	46.9 47.9	43.3 53.3
Cifti [28]	Turkey	Lumbar discectomy	20 mL 0.25% bupivacaine	No block	Fentanyl	Study Control	30 30	45.9 44.1	53.3 50
Ni [29]	China	Lumbar spine fusion surgery	20 ml of 0.375% ropivacaine	No block	Sufentanil	Study Control	67 67	51 54	56.7 50.7
Bicak [12]	Turkey	Lumbar disc surgery	20 mL 0.25% bupivacaine	Wound infiltration	Tramadol	Study Control	21 21	43.2 48.6	61.9 52.3
Eltaher [13]	Egypt	Lumbar spine surgery	20 mL 0.25% bupivacaine	Sham block	Morphine	Study Control	30 30	NR	NR
Wang [30]	China	Lumbar spine fusion surgery	30 ml of 0.375% ropivacaine	No block	Sufentanil and flurbiprofen	Study Control	102 102	52.7 55.7	58 50
Pavithran [11]	India	Posterior lumbar spine fusion	25 ml of a mixture of 40 ml of 0.375% ropivacaine, 10 ml of 2% lignocaine and 4 mg dexamethasone	Wound infiltration	Tramadol	Study Control	35 35	53.2 51.2	55.6 28.6

*PCA* Patient-controlled analgesia; *ESPB* Erector spinae plane block; *TLIP* Thoracolumbar interfascial plane block; *NR* Not reported

### Pain scores

**Fig. 2:**
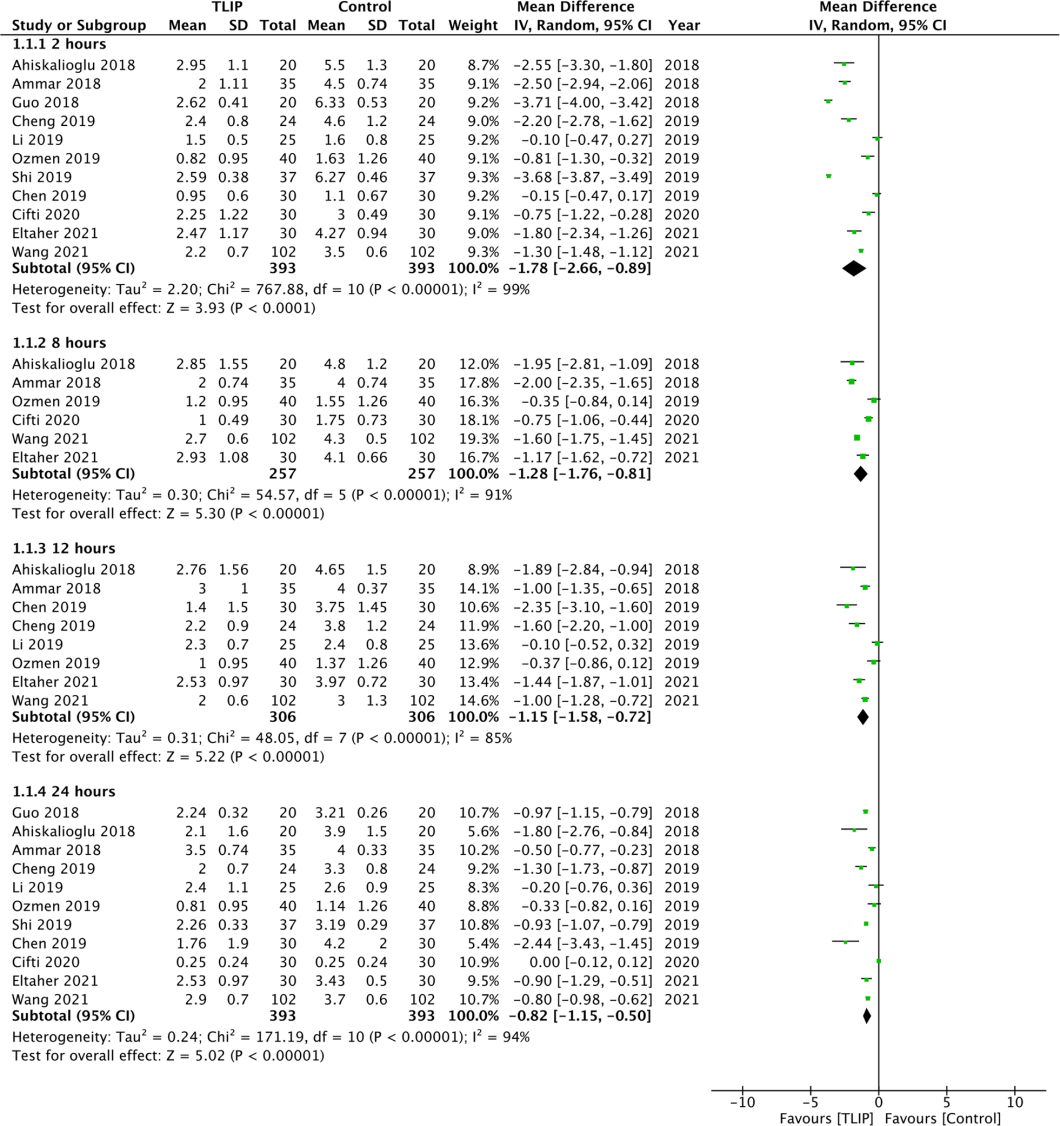
Meta-analysis of pain scores at rest between TLIP and no block/sham block

**Fig. 3:**
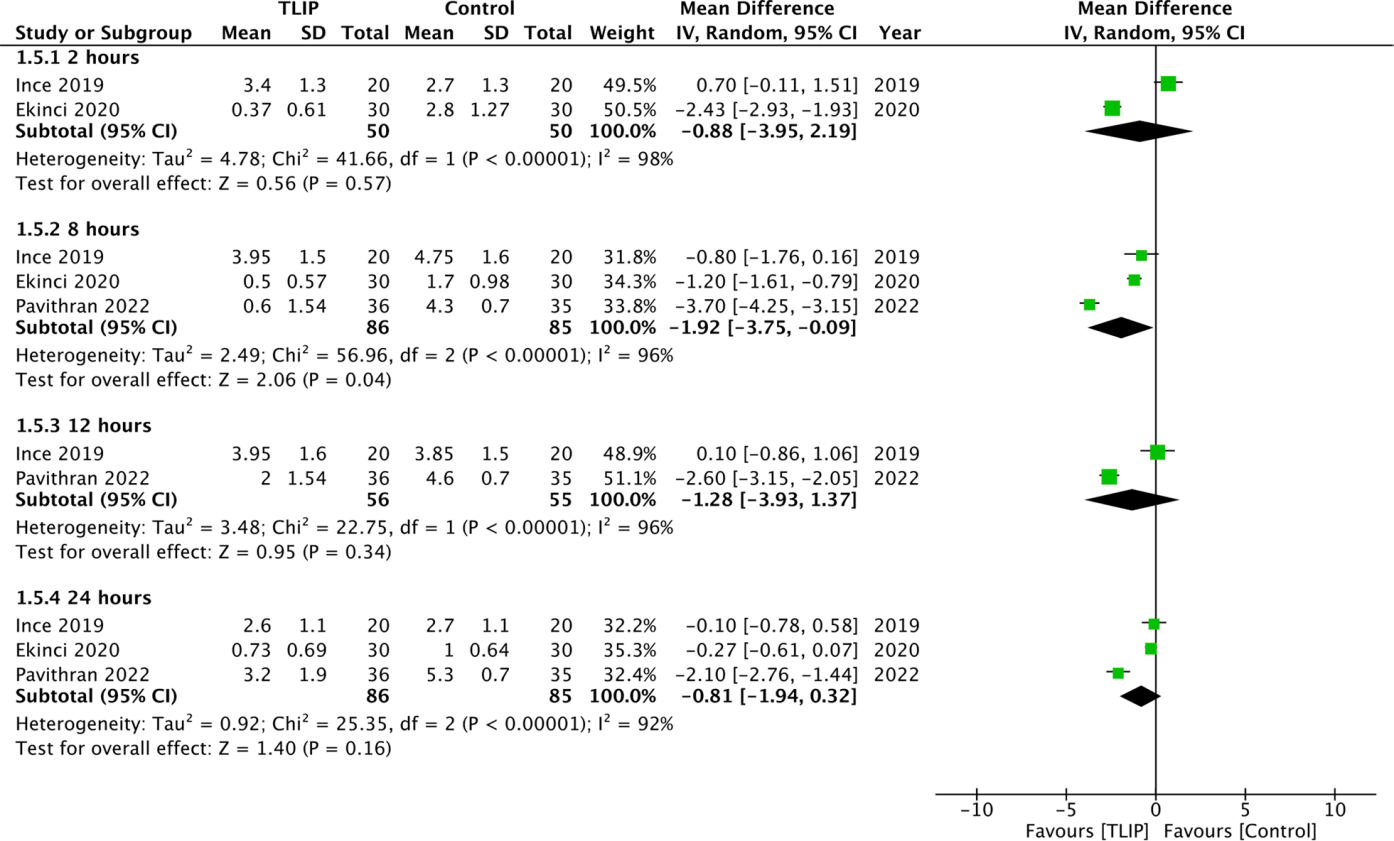
Meta-analysis of pain scores at rest between TLIP and wound infiltration

Comparing TLIP with no block or sham block, the meta-analysis showed a significant reduction of pain scores at rest at 2 h (MD: − 1.78 95% CI − 2.66, − 0.89 *I*^2^ = 99%), 8 h (MD: − 1.28 95% CI − 1.76, − 0.81 *I*^2^ = 91%), 12 h (MD: − 1.15 95% CI − 1.58, − 0.72 *I*^2^ = 85%) and 24 h (MD: − 0.82 95% CI − 1.15, − 0.50 *I*^2^ = 94%) (Fig. 2). Sensitivity analysis did not change these results. Minimal data showed a significant difference in pain scores at rest between TLIP and wound infiltration group at 8 h (MD: − 1.92 95% CI − 3.75, − 0.09 *I*^2^ = 96%) but not at 2 h (MD: − 0.88 95% CI − 3.95, − 2.19 *I*^2^ = 98%), 12 h (MD: − 1.28 95% CI − 3.93, − 1.37 *I*^2^ = 96%) and 24 h (MD: − 0.81 95% CI − 1.94, 0.32 *I*^2^ = 92%) (Fig. 3).

**Fig. 4:**
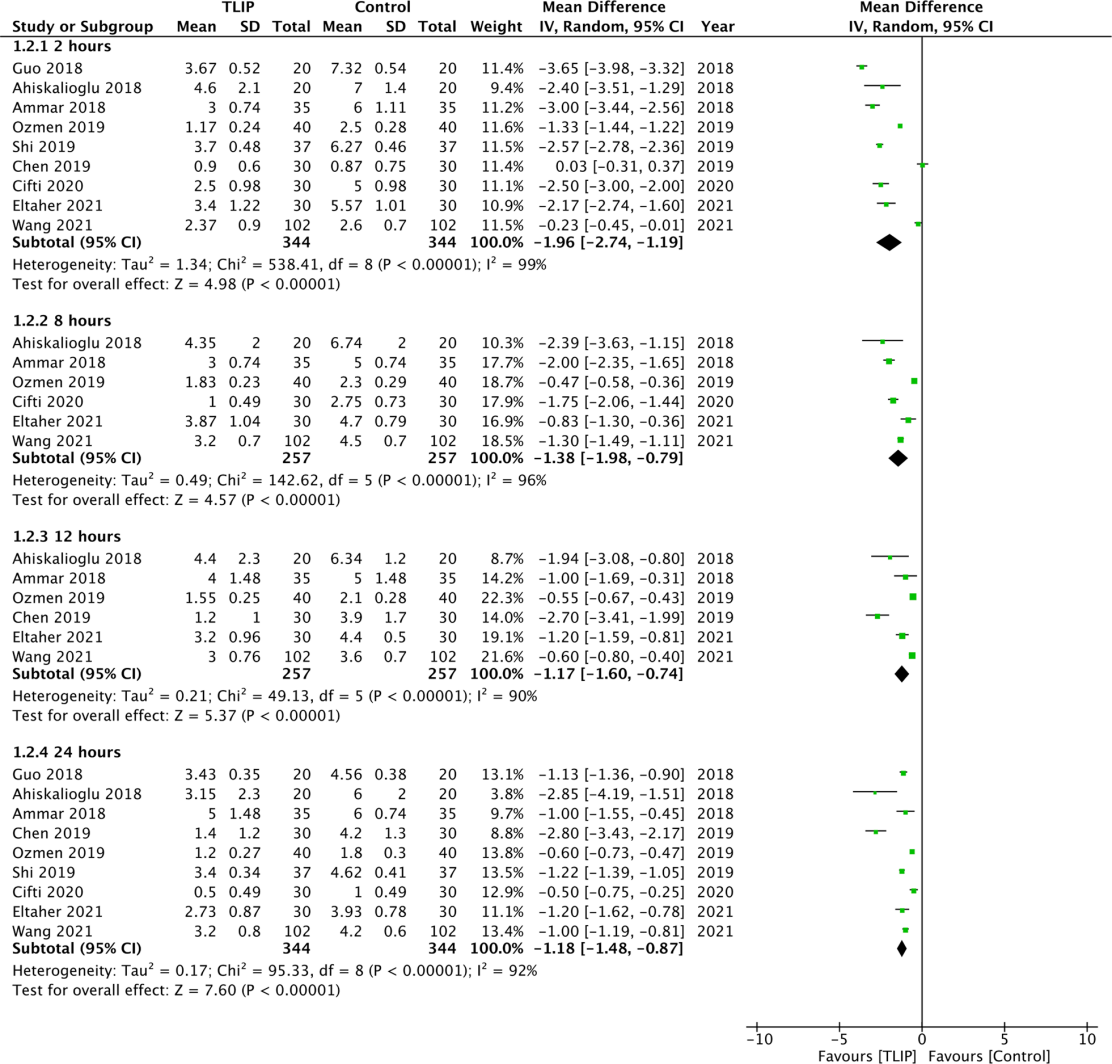
Meta-analysis of pain scores at movement between TLIP and no block/sham block

Similarly, a significant reduction was noted in pain scores at rest at 2 h (MD: − 1.96 95% CI − 2.74, − 1.19 *I*^2^ = 99%), 8 h (MD: − 1.38 95% CI − 1.98, − 0.79 *I*^2^ = 96%), 12 h (MD: − 1.17 95% CI − 1.60, − 0.74 *I*^2^ = 90%) and 24 h (MD: − 1.18 95% CI − 1.48, − 0.87 *I*^2^ = 92%) when comparing TLIP and no block/sham block (Fig. 4). The significance of effect size did not change on sensitivity analysis. Sufficient data were not available for a meta-analysis comparing pain on movement between TLIP and wound infiltration.

### Analgesic consumption and PONV

**Fig. 5:**
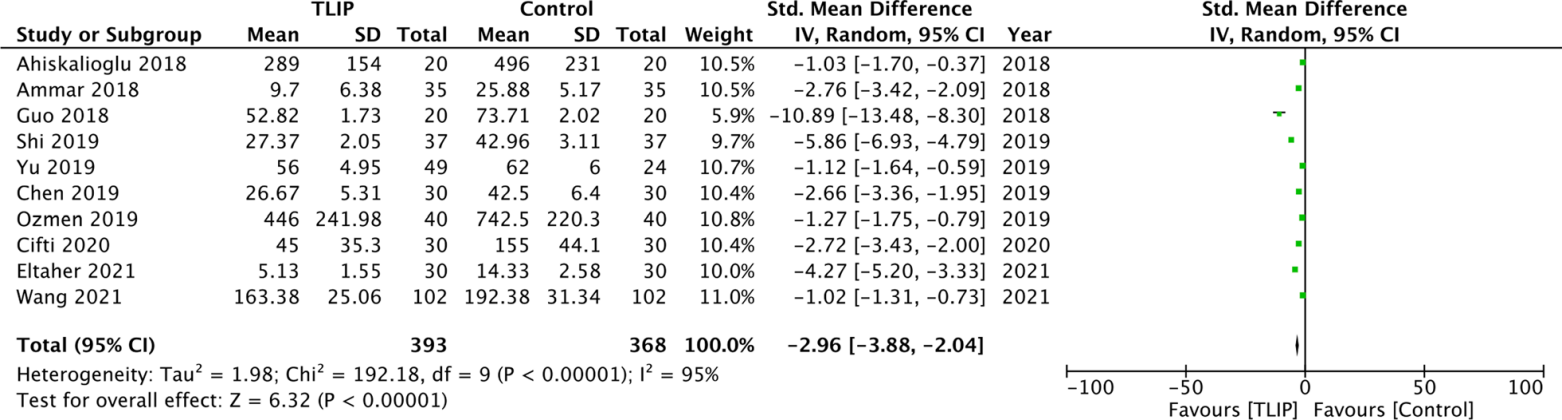
Meta-analysis of total analgesic consumption between TLIP and no block/sham block

**Fig. 6:**
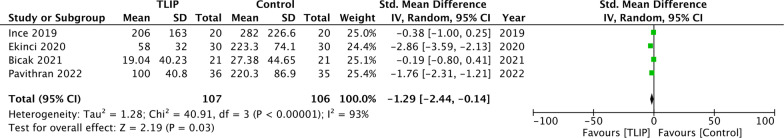
Meta-analysis of total analgesic consumption between TLIP and wound infiltration

Pooled analysis showed a statistically significant reduction in total postoperative analgesic consumption with TLIP as compared to no block/sham block (SMD: − 2.96 95% CI − 3.88, − 2.04 *I*^2^ = 95%) (Fig. 5). The results remained significant on the sequential exclusion of studies. The funnel plot indicated no publication bias and a small effect size (Additional file 2: Fig. S1). Combined analysis of all four studies using local anesthetic wound infiltration showed a statistically significant reduction in total postoperative analgesic consumption with TLIP in patients undergoing lumbar spinal surgeries (SMD: − 1.29 95% CI − 2.44, − 0.14 *I*^2^ = 93%) (Fig. 6).

**Fig. 7:**
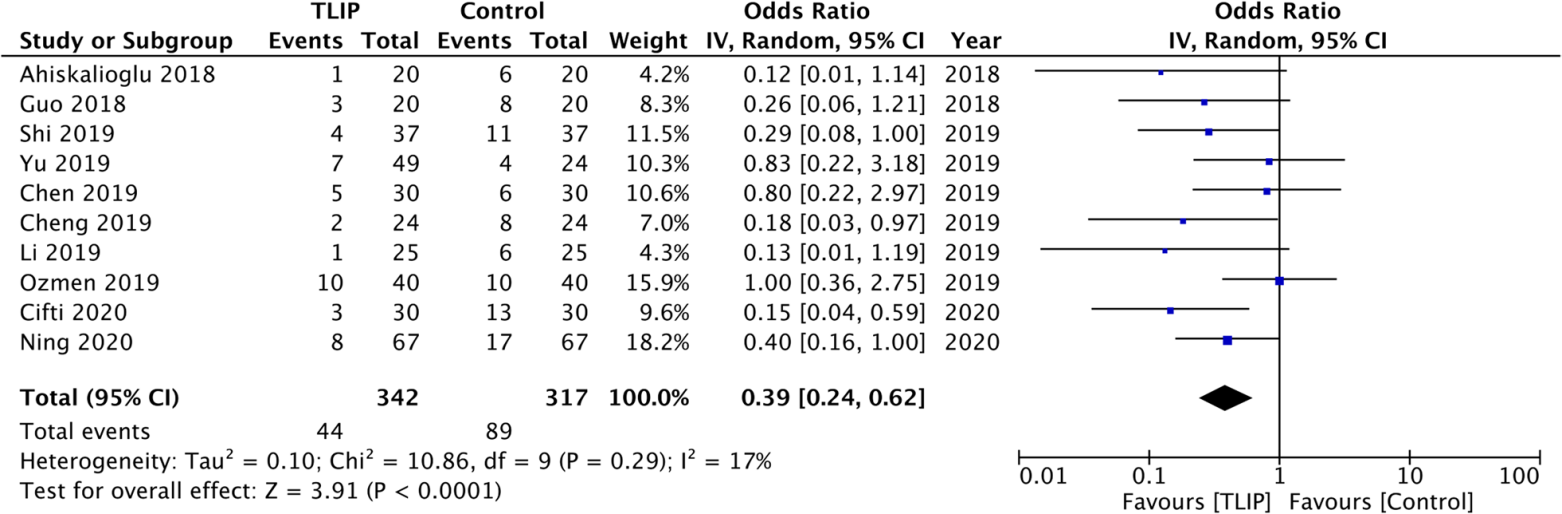
Meta-analysis of PONV between TLIP and no block/sham block

Meta-analysis also showed significantly reduced odds of PONV with TLIP as compared to no block/sham block (OR: 0.39 95% CI 0.24, 0.62 *I*^2^ = 17%) (Fig. 7). There was no change in the results on sensitivity analysis. Sufficient data were not available for a meta-analysis comparing PONV between TLIP and wound infiltration.

GRADE assessment of the evidence is shown in Additional file 3: Table S2. The certainty of the evidence was moderate for all outcomes. The certainty of the evidence for pain scores between TLIP and wound infiltration was not examined due to scarce data.

### Risk of bias

**Fig. 8:**
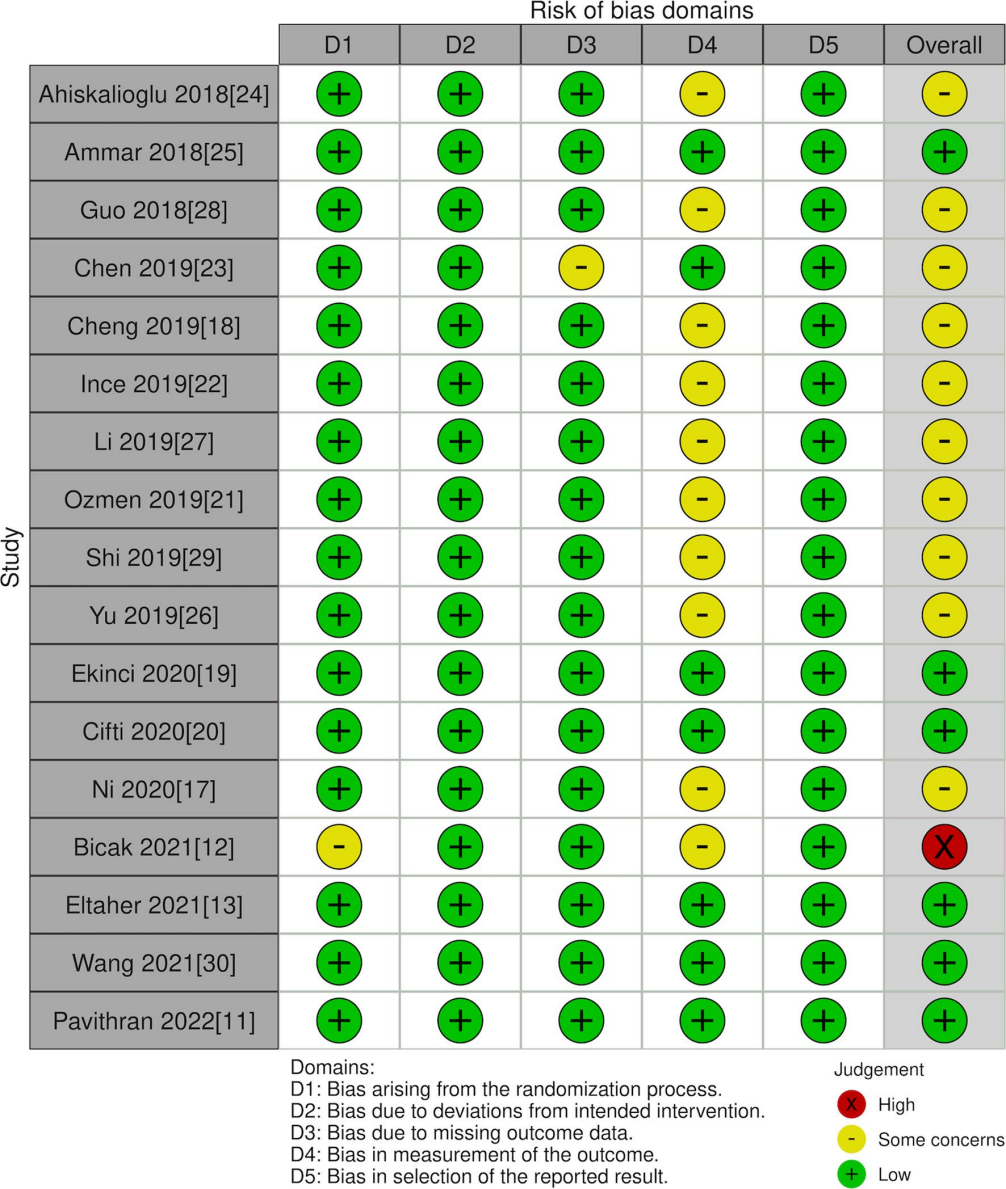
Risk of bias among studies

The risk of bias in each study as per the reviewer’s opinion is shown in Table 2. Six RCTs had a low risk, 10 had some concerns, and one had a high risk of bias (Fig. 8).

**Table 2:** Risk of bias analysis

Study	Randomization process	Deviation from intended intervention	Missing outcome data	Measurement of outcomes	Selection of reported result	Overall risk of bias
Ahiskalioglu [17]	Low risk	Low risk	Low risk	Some concerns	Low risk	Some concerns
Ammar [18]	Low risk	Low risk	Low risk	Low risk	Low risk	Low risk
Guo [19]	Low risk	Low risk	Low risk	Some concerns	Low risk	Some concerns
Chen [20]	Low risk	Low risk	Some concerns	Low risk	Low risk	Some concerns
Cheng [21]	Low risk	Low risk	Low risk	Some concerns	Low risk	Some concerns
Ince [22]	Low risk	Low risk	Low risk	Some concerns	Low risk	Some concerns
Li [23]	Low risk	Low risk	Low risk	Some concerns	Low risk	Some concerns
Ozmen [24]	Low risk	Low risk	Low risk	Some concerns	Low risk	Some concerns
Shi [25]	Low risk	Low risk	Low risk	Some concerns	Low risk	Some concerns
Yu [26]	Low risk	Low risk	Low risk	Some concerns	Low risk	Some concerns
Ekinci [27]	Low risk	Low risk	Low risk	Low risk	Low risk	Low risk
Cifti [28]	Low risk	Low risk	Low risk	Low risk	Low risk	Low risk
Ni [29]	Low risk	Low risk	Low risk	Some concerns	Low risk	Some concerns
Bicak [12]	Some concerns	Low risk	Low risk	Some concerns	Low risk	High risk
Eltaher [13]	Low risk	Low risk	Low risk	Low risk	Low risk	Low risk
Wang [30]	Low risk	Low risk	Low risk	Low risk	Low risk	Low risk
Pavithran [11]	Low risk	Low risk	Low risk	Low risk	Low risk	Low risk

## Discussion

This updated meta-analysis has shown that TLIP is effective in reducing pain scores at rest as well as movement in the first 24 h after surgery as compared to no block. However, pain scores were not reduced at all time points when compared with wound infiltration of the surgical site with local anesthetics. Nevertheless, TLIP was able to reduce total analgesic consumption when compared to both, with no block and wound infiltration. PONV was also significantly reduced with TLIP.

Postoperative pain control after lumbar surgeries has been a topic of interest for researchers in the past decade [31]. Indeed, poor pain control can not only impact patient satisfaction but also delay rehabilitation and increase healthcare costs. In this context, there has been a search for optimal regional anesthetic techniques which can provide sufficient analgesia in the initial postoperative period with a concurrent decrease in opioid use in patients undergoing lumbar surgeries. One such novel technique, i.e., the TLIP block, was first tested by Hand et al. [7] in 2015. The block was to target the dorsal rami of the thoracolumbar nerves similar to the transversus abdominis plane (TAP) block which blocks the ventral rami during their passage via the paraspinal muscles. Since the TAP block is effective in providing analgesia in those undergoing lower abdominal surgeries [32], the authors postulated that a similar effect could be achieved for lumbar surgeries by blocking the dorsal branches. The anesthetic agent in TLIP is injected in the fascial plane between the multifidus and longissimus muscles of the thoracolumbar spine with anesthesia achieved in the midline at the level of the injection [7]. While its effect was demonstrated in 10 health volunteers by Hand et al. [7], its clinical efficacy has been a subject of research of many RCTs with variable sample sizes and results. Considering the recent discovery of this novel block, it is important to generate high-quality evidence on its analgesic efficacy to recommend clinical use.

Our meta-analysis of 17 RCTs has generated the most updated and detailed evidence on the effectiveness of TLIP for patients undergoing lumbar surgery. It was found that TLIP was effective in reducing pain scores measured on a 10-point scale at 2, 8, 12, and 24 h when compared with no or sham blocks. Reduction in pain was noted both during rest and movement. Pain reduction at rest was noted to be highest in the initial period, i.e., at 2 h with a 1.78 point decrease in pain scores with a gradual reduction in efficacy to a 0.82 point reduction at 24 h. Similarly, pain scores at movement followed an identical pattern with the highest efficacy at 2 h (1.96 point reduction) and the lowest at 24 h (1.18 point reduction). The results of individual studies were mostly consistent favoring TLIP with no change in significance on the removal of any trial thereby increasing the credibility of the evidence.

Wound infiltration of local anesthetics is commonly practiced in many healthcare setups for pain control after lumbar surgery. However, the clinical significance of this practice has been questionable with limited studies showing a small reduction in pain scores. Also, no clinically significant reduction in opioid consumption has been noted with such practice [33]. Since some of the trials compared TLIP with wound infiltration, we conducted a separate analysis to compare these two groups. On comparison, pain scores were found to be reduced at only 8 h with TLIP with no statistically significant difference at other time points. This could be due to the scarce data and the limited analgesic effect offered by the local anesthetics in the initial postoperative period. However, total analgesic consumption was significantly reduced with TLIP when compared to both no/sham block and wound infiltration, albeit with a smaller difference with the latter, thereby confirming the analgesic efficacy of TLIP in the initial postoperative period. The significant reduction in PONV also confirms the reduction in opioid use with TLIP block.

Our results concur with prior meta-analyses [8, 9] on TLIP but with significant differences. Ye et al. [9] in a meta-analysis of nine studies noted a significant reduction in pain scores and total analgesic consumption with TLIP as compared to no or sham block. They also noted identical pain scores with TLIP and wound infiltration but with a significant reduction in total analgesic consumption with the former. Similarly, Hu et al. [8] pooled data from nine RCTs to show a significant reduction in pain scores (at rest and movement), total analgesic consumption, and PONV with TLIP as compared to the control. The current review including 17 trials is a significant update from the previous reviews [8, 9] by significantly increasing the power of the analysis. Secondly, retracted and overlapping studies [10, 34] included in the review of Ye et al. [9] were also omitted from the review to increase the veracity of the evidence.

The current review has some limitations. Firstly, the high heterogeneity is a cause of concern. All of the analyses, except for PONV had extremely high inter-study heterogeneity probably due to differences in the study subjects, the type of baseline anesthetic protocols, the anesthetic agent, and the analgesics used. Hence, the results are to be construed with caution. Secondly, data on other variables like the need for rescue analgesia and other complications were scarcely reported and hence a quantitative analysis was not conducted. Thirdly, limited studies were available for comparison of TLIP with wound infiltration and many of the outcomes like pain on movement and PONV could not be analyzed due to lack of data. Also, in the protocol published on PROSPERO, it was initially planned to compare TLIP with erector spinae plane blocks, however, the same was abandoned due to want of studies. Fourthly, only six of the included studies had low risk of bias and the overall quality of studies was low-moderate. The risk of bias in the included studies downgraded the overall certainty of evidence. Lastly, most of the studies were from a limited number of countries, which prohibits the generalizability of results.

## Conclusion

Moderate quality evidence suggests that TLIP blocks are effective in pain control after lumbar spinal surgeries. TLIP reduces pain scores at rest and movement for up to 24 h and reduces total analgesic consumption and the incidence of PONV. However, evidence of its efficacy as compared to wound infiltration of local anesthetics is scarce. Because of low to moderate quality of the primary studies and marked heterogeneity of the pooled results, the benefit of TLIP should be interpreted with cautions. Further trials are needed to obtain evidence on the efficacy of TLIP vs wound infiltration and erector spinae plane blocks.

## Supplementary Information

**Additional file 1: Table S1.** Search strategy.

**Additional file 2: Fig. S1.** Funnel plot to assess publication bias.

**Additional file 3: Table S2.** GRADE assessment of evidence.

## Data Availability

Not applicable.
