# Supplementary material for: The efficacy of thoracolumbar interfascial plane block for lumbar spinal surgeries: a systematic review and meta-analysis
Source: J Orthop Surg Res. 2023 Apr 25;18:318. doi: 10.1186/s13018-023-03798-2 (PMC10127357; doi:10.1186/s13018-023-03798-2)
Supplement: Supplementary file 1 — Additional file 1: Table S1. Search strategy. [file 13018_2023_3798_MOESM1_ESM.docx]

Supplementary Table 1: Search strategy

| **Query** | **Search Details** |
| --- | --- |
| ((lumbar surgery) AND (analgesia)) AND (randomised controlled trial) | ("lumbarised"[All Fields] OR "lumbarization"[All Fields] OR "lumbarized"[All Fields] OR "lumbars"[All Fields] OR "lumbosacral region"[MeSH Terms] OR ("lumbosacral"[All Fields] AND "region"[All Fields]) OR "lumbosacral region"[All Fields] OR "lumbar"[All Fields]) AND ("surgery"[MeSH Subheading] OR "surgery"[All Fields] OR "surgical procedures, operative"[MeSH Terms] OR ("surgical"[All Fields] AND "procedures"[All Fields] AND "operative"[All Fields]) OR "operative surgical procedures"[All Fields] OR "general surgery"[MeSH Terms] OR ("general"[All Fields] AND "surgery"[All Fields]) OR "general surgery"[All Fields] OR "surgery s"[All Fields] OR "surgerys"[All Fields] OR "surgeries"[All Fields]) AND ("analgesia"[MeSH Terms] OR "analgesia"[All Fields] OR "analgesias"[All Fields]) AND ("randomized controlled trial"[Publication Type] OR "randomized controlled trials as topic"[MeSH Terms] OR "randomised controlled trial"[All Fields] OR "randomized controlled trial"[All Fields]) |
| (((Spine) OR (lumbar)) OR (spinal)) AND (TLIP block) | ("spine"[MeSH Terms] OR "spine"[All Fields] OR "spines"[All Fields] OR "spine s"[All Fields] OR ("lumbarised"[All Fields] OR "lumbarization"[All Fields] OR "lumbarized"[All Fields] OR "lumbars"[All Fields] OR "lumbosacral region"[MeSH Terms] OR ("lumbosacral"[All Fields] AND "region"[All Fields]) OR "lumbosacral region"[All Fields] OR "lumbar"[All Fields]) OR ("spinal"[All Fields] OR "spinalization"[All Fields] OR "spinalized"[All Fields] OR "spinally"[All Fields] OR "spinals"[All Fields])) AND ("TLIP"[All Fields] AND ("block"[All Fields] OR "blocked"[All Fields] OR "blocking"[All Fields] OR "blockings"[All Fields] OR "blocks"[All Fields])) |
| (((Spine) OR (lumbar)) OR (spinal)) AND (thoracolumbar interfascial plane block) | ("spine"[MeSH Terms] OR "spine"[All Fields] OR "spines"[All Fields] OR "spine s"[All Fields] OR ("lumbarised"[All Fields] OR "lumbarization"[All Fields] OR "lumbarized"[All Fields] OR "lumbars"[All Fields] OR "lumbosacral region"[MeSH Terms] OR ("lumbosacral"[All Fields] AND "region"[All Fields]) OR "lumbosacral region"[All Fields] OR "lumbar"[All Fields]) OR ("spinal"[All Fields] OR "spinalization"[All Fields] OR "spinalized"[All Fields] OR "spinally"[All Fields] OR "spinals"[All Fields])) AND ("thoracolumbar"[All Fields] AND "interfascial"[All Fields] AND ("aircraft"[MeSH Terms] OR "aircraft"[All Fields] OR "plane"[All Fields] OR "planes"[All Fields]) AND ("block"[All Fields] OR "blocked"[All Fields] OR "blocking"[All Fields] OR "blockings"[All Fields] OR "blocks"[All Fields])) |
