# Supplementary figures and images for: The efficacy of thoracolumbar interfascial plane block for lumbar spinal surgeries: a systematic review and meta-analysis
Source: J Orthop Surg Res. 2023 Apr 25;18:318. doi: 10.1186/s13018-023-03798-2 (PMC10127357; doi:10.1186/s13018-023-03798-2)

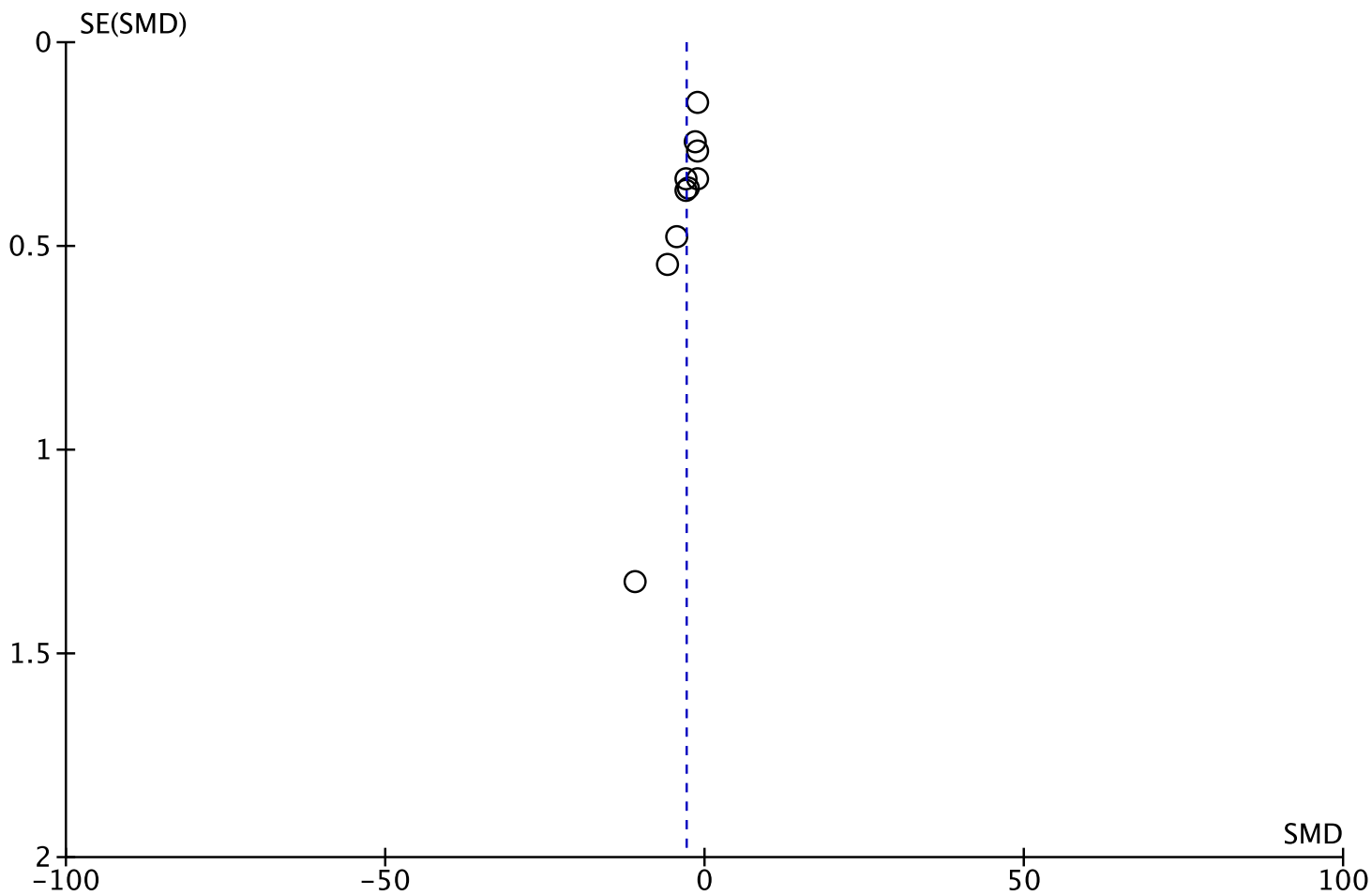

Supplement: Supplementary file 2 — Additional file 2: Fig. S1. Funnel plot to assess publication bias. [file 13018_2023_3798_MOESM2_ESM.pdf]
