# Supplementary material for: The efficacy of thoracolumbar interfascial plane block for lumbar spinal surgeries: a systematic review and meta-analysis
Source: J Orthop Surg Res. 2023 Apr 25;18:318. doi: 10.1186/s13018-023-03798-2 (PMC10127357; doi:10.1186/s13018-023-03798-2)
Supplement: Supplementary file 3 — Additional file 3: Table S2. GRADE assessment of evidence. [file 13018_2023_3798_MOESM3_ESM.docx]

| **Supplementary Table 2: GRADE assessment of evidence** | | | | | | | | | | | |
| --- | --- | --- | --- | --- | --- | --- | --- | --- | --- | --- | --- |
| **Certainty assessment** | | | | | | | **Summary of findings** | | | | |
| **Participants (studies) Follow-up** | **Risk of bias** | **Inconsistency** | **Indirectness** | **Imprecision** | **Publication bias** | **Overall certainty of evidence** | **Study event rates (%)** | | **Relative effect (95% CI)** | **Anticipated absolute effects** | |
|  |  |  |  |  |  |  | **With placebo** | **With New Comparison** |  | **Risk with placebo** | **Risk difference with New Comparison** |
| **Pain rest - 2 hours** | | | | | | | | | | | |
| 786 (11 RCTs) | serious^a^ | not serious | not serious | not serious | none | ⨁⨁⨁◯ Moderate | 393 | 393 | - | The mean pain rest - 2 hours was **0** | MD **1.78 lower** (2.66 lower to 0.89 lower) |
| **Pain rest - 8 hours** | | | | | | | | | | | |
| 514 (6 RCTs) | serious^a^ | not serious | not serious | not serious | none | ⨁⨁⨁◯ Moderate | 257 | 257 | - | The mean pain rest - 8 hours was **0** | MD **1.28 lower** (1.77 lower to 0.81 lower) |
| **Pain rest - 12 hours** | | | | | | | | | | | |
| 614 (8 RCTs) | serious^a^ | not serious | not serious | not serious | none | ⨁⨁⨁◯ Moderate | 306 | 306 | - | The mean pain rest - 12 hours was **0** | MD **1.15 lower** (1.58 lower to 0.72 lower) |
| **Pain rest - 24 hours** | | | | | | | | | | | |
| 786 (11 RCTs) | serious^a^ | not serious | not serious | not serious | none | ⨁⨁⨁◯ Moderate | 393 | 393 | - | The mean pain rest - 24 hours was **0** | MD **0.82 lower** (1.15 lower to 0.5 lower) |
| **Pain movement - 2 hours** | | | | | | | | | | | |
| 688 (9 RCTs) | serious^a^ | not serious | not serious | not serious | none | ⨁⨁⨁◯ Moderate | 344 | 344 | - | The mean pain movement - 2 hours was **0** | MD **1.96 lower** (2.74 lower to 1.19 lower) |
| **Pain movement - 8 hours** | | | | | | | | | | | |
| 514 (6 RCTs) | not serious | not serious | not serious | not serious | none | ⨁⨁⨁⨁ High | 257 | 257 | - | The mean pain movement - 8 hours was **0** | MD **1.38 lower** (1.98 lower to 0.79 lower) |
| **Pain movement - 12 hours** | | | | | | | | | | | |
| 514 (6 RCTs) | serious^a^ | not serious | not serious | not serious | none | ⨁⨁⨁◯ Moderate | 257 | 257 | - | The mean pain movement - 12 hours was **0** | MD **1.17 lower** (1.60 lower to 0.74 lower) |
| **Pain movement - 24 hours** | | | | | | | | | | | |
| 688 (9 RCTs) | serious^a^ | not serious | not serious | not serious | none | ⨁⨁⨁◯ Moderate | 344 | 344 | - | The mean pain movement - 24 hours was **0** | MD **1.18 lower** (1.48 lower to 0.87 lower) |
| **PCA: TLIP vs no block/sham block** | | | | | | | | | | | |
| 761 (10 RCTs) | serious^a^ | not serious | not serious | not serious | none | ⨁⨁⨁◯ Moderate | 393 | 368 | - | - | SMD **2.96 lower** (3.88 lower to 2.04 lower) |
| **PONV: TLIP vs no block/sham block** | | | | | | | | | | | |
| 659 (10 RCTs) | serious^a^ | not serious | not serious | not serious | none | ⨁⨁⨁◯ Moderate | 44/342 (12.9%) | 89/317 (28%) | **OR 0.39** (0.24 to 0.62) | 282 per 1,000 | **155 fewer per 1,000** (from 196 fewer to 96 fewer) |
| **PCA: TLIP vs wound infiltration** | | | | | | | | | | | |
| 213 (4 RCTs) | serious^a^ | not serious | not serious | not serious | none | ⨁⨁⨁◯ Moderate | 106 | 107 | - | - | SMD **1.29 lower** (2.44 lower to 0.14 lower) |

**CI:** confidence interval; **MD:** mean difference; **OR:** odds ratio; **SMD:** standardised mean difference

#### Explanations

a. Some RCTs with "some concerns" in the overall risk of bias analysis
